# Differential and longitudinal immune gene patterns associated with reprogrammed microenvironment and viral mimicry in response to neoadjuvant radiotherapy in rectal cancer

**DOI:** 10.1136/jitc-2020-001717

**Published:** 2021-03-07

**Authors:** Anna Wilkins, Elisa Fontana, Gift Nyamundanda, Chanthirika Ragulan, Yatish Patil, David Mansfield, Jennifer Kingston, Fiona Errington-Mais, Daniel Bottomley, Katharina von Loga, Hannah Bye, Paul Carter, Emma Tinkler-Hundal, Arish Noshirwani, Jessica Downs, Magnus Dillon, Sandra Demaria, David Sebag-Montefiore, Kevin Harrington, Nick West, Alan Melcher, Anguraj Sadanandam

**Affiliations:** 1 Division of Radiotherapy and Imaging, Institute of Cancer Research, London, UK; 2 The Francis Crick Institute, London, UK; 3 Division of Molecular Pathology, Institute of Cancer Research, London, UK; 4 Current Affiliation: Sarah Cannon Research Institute, London, UK; 5 Leeds Institute of Medical Research at St. James’s, University of Leeds, Leeds, UK; 6 The Royal Marsden Hospital, London, UK; 7 Division of Cancer Biology, Institute of Cancer Research, London, UK; 8 Weill Cornell Medicine, New York, NY, USA

**Keywords:** gene expression profiling, gastrointestinal neoplasms, immunotherapy, tumor microenvironment, macrophages

## Abstract

**Background:**

Rectal cancers show a highly varied response to neoadjuvant radiotherapy/chemoradiation (RT/CRT) and the impact of the tumor immune microenvironment on this response is poorly understood. Current clinical tumor regression grading systems attempt to measure radiotherapy response but are subject to interobserver variation. An unbiased and unique histopathological quantification method (change in tumor cell density (ΔTCD)) may improve classification of RT/CRT response. Furthermore, immune gene expression profiling (GEP) may identify differences in expression levels of genes relevant to different radiotherapy responses: (1) at baseline between poor and good responders, and (2) longitudinally from preradiotherapy to postradiotherapy samples. Overall, this may inform novel therapeutic RT/CRT combination strategies in rectal cancer.

**Methods:**

We generated GEPs for 53 patients from biopsies taken prior to preoperative radiotherapy. TCD was used to assess rectal tumor response to neoadjuvant RT/CRT and ΔTCD was subjected to k-means clustering to classify patients into different response categories. Differential gene expression analysis was performed using statistical analysis of microarrays, pathway enrichment analysis and immune cell type analysis using single sample gene set enrichment analysis. Immunohistochemistry was performed to validate specific results. The results were validated using 220 pretreatment samples from publicly available datasets at metalevel of pathway and survival analyses.

**Results:**

ΔTCD scores ranged from 12.4% to −47.7% and stratified patients into three response categories. At baseline, 40 genes were significantly upregulated in poor (n=12) versus good responders (n=21), including myeloid and stromal cell genes. Of several pathways showing significant enrichment at baseline in poor responders, epithelial to mesenchymal transition, coagulation, complement activation and apical junction pathways were validated in external cohorts. Unlike poor responders, good responders showed longitudinal (preradiotherapy vs postradiotherapy samples) upregulation of 198 immune genes, reflecting an increased T-cell-inflamed GEP, type-I interferon and macrophage populations. Longitudinal pathway analysis suggested viral-like pathogen responses occurred in post-treatment resected samples compared with pretreatment biopsies in good responders.

**Conclusion:**

This study suggests potentially druggable immune targets in poor responders at baseline and indicates that tumors with a good RT/CRT response reprogrammed from immune “cold” towards an immunologically “hot” phenotype on treatment with radiotherapy.

## Introduction

More than 700 000 new cases and 300 000 deaths of rectal cancer per annum were estimated in the Global **Cancer** Incidence, Mortality and Prevalence (GLOBOCAN) 2018 report.^1^ These numbers are expected to rise, especially in young adults.^1 2^ Neoadjuvant radiotherapy/chemoradiation (RT/CRT) is a recommended strategy for the majority of patients presenting with locally advanced adenocarcinoma of the rectum.^3^ Either short-course radiotherapy (25 Gy in 5 fractions over 5 days) (SCRT) or long-course chemoradiation (45 Gy in 25 fractions over 5 weeks concomitant with fluoropyrimidine-based chemotherapy) (LCRT) are established regimens with similar (local) tumor control benefits.^3^

Only 20% of patients show a complete pathological response (pathCR) following RT/CRT and this is associated with significantly better survival outcomes.^3 4^ In the remaining 80%, a wide variation in response is observed. Some intensified neoadjuvant regimens, which include the addition of oxaliplatin, have shown improved rates of pathCR and organ preservation.^5 6^ However, a number of other regimens have failed to meet predefined clinical trial endpoints,^4^ driving the search for new therapeutic strategies.

At present, the standard for the assessment of rectal tumor response to neoadjuvant therapy is the tumor regression grade (TRG). A number of TRG systems exist, of which the American Joint Committee on Cancer (AJCC) TRG system has shown superior prediction of survival outcomes.^7^ However, the AJCC TRG has only four categories, meaning that considerable variation in radiotherapy response may occur within a single score. In addition, all TRG systems are limited by the subjectivity inherent to manual scoring and differences in interobserver agreement.^3^ Evaluation of tumor cell density (TCD) involves point quantification of 300 individual tumor or stromal cells in H&E stained tumor sections.^8 9^ TCD is similar to TRG in that it assesses the relative proportion of tumor and stroma cells; however, in contrast to TRG, TCD does this using a linear and objective measurement. The use of TCD may therefore enable increased precision and detection of smaller differences in radiotherapy response than TRG.

The role of the tumor immune microenvironment in radioresponsiveness is increasingly recognized.^10^ However, a clear understanding of the immune biology underlying radioresponsiveness in rectal cancer is lacking. Microsatellite instability occurs in less than 2% of rectal tumors,^11^ and appears to be associated with greater responsiveness to chemoradiotherapy than neoadjuvant fluorouracil/oxaliplatin, for reasons that are not fully understood.^12^ Furthermore, aspects of tumor biology associated with response to novel immunomodulatory agents, such as tumor mutational burden and neoantigen load, do not show an association with radiotherapy response.^11 13^

Very few studies have reported immune gene expression changes associated with radioresponsiveness, or potential personalized radiotherapy approaches based on the underlying tumor immune microenvironment in rectal cancer.^13 14^ This is partly because of the paucity of rectal cancer-specific transcriptional datasets with paired preradiotherapy and postradiotherapy samples and well-annotated radiotherapy responses. Published reports that do exist have typically used a variety of different gene expression technologies and often evaluated non-overlapping gene sets. This has restricted their ability to validate novel findings in external cohorts and it has therefore been difficult to reach a consensus view on the gene signatures driving radiotherapy resistance in rectal cancer.

This study aimed to characterize the key immunological gene expression profiles (GEP), pathways and cell types associated with response or resistance to neoadjuvant RT/CRT at baseline and longitudinally (changes evolving during treatment). In the future, this characterization could inform the development of potential gene expression biomarkers (although not within the scope of the current manuscript) and novel immunotherapy combination strategies to improve tumor response to radiotherapy in rectal cancer.

## Methods

### Study population

Pretreatment biopsy and post-treatment resection specimen archival blocks from patients who received SCRT or LCRT atNorth East – York Research Ethics Committee were retrieved (Research Ethics Committee No 08/H0903/62). Clinicopathological characteristics of the patients were collected. Patients with a non-standard interval between the end of radiation and surgery (longer than 20 and 80 days for SCRT and LCRT, respectively) were excluded.

### Definition of response to neoadjuvant therapy

**Figure 1:**
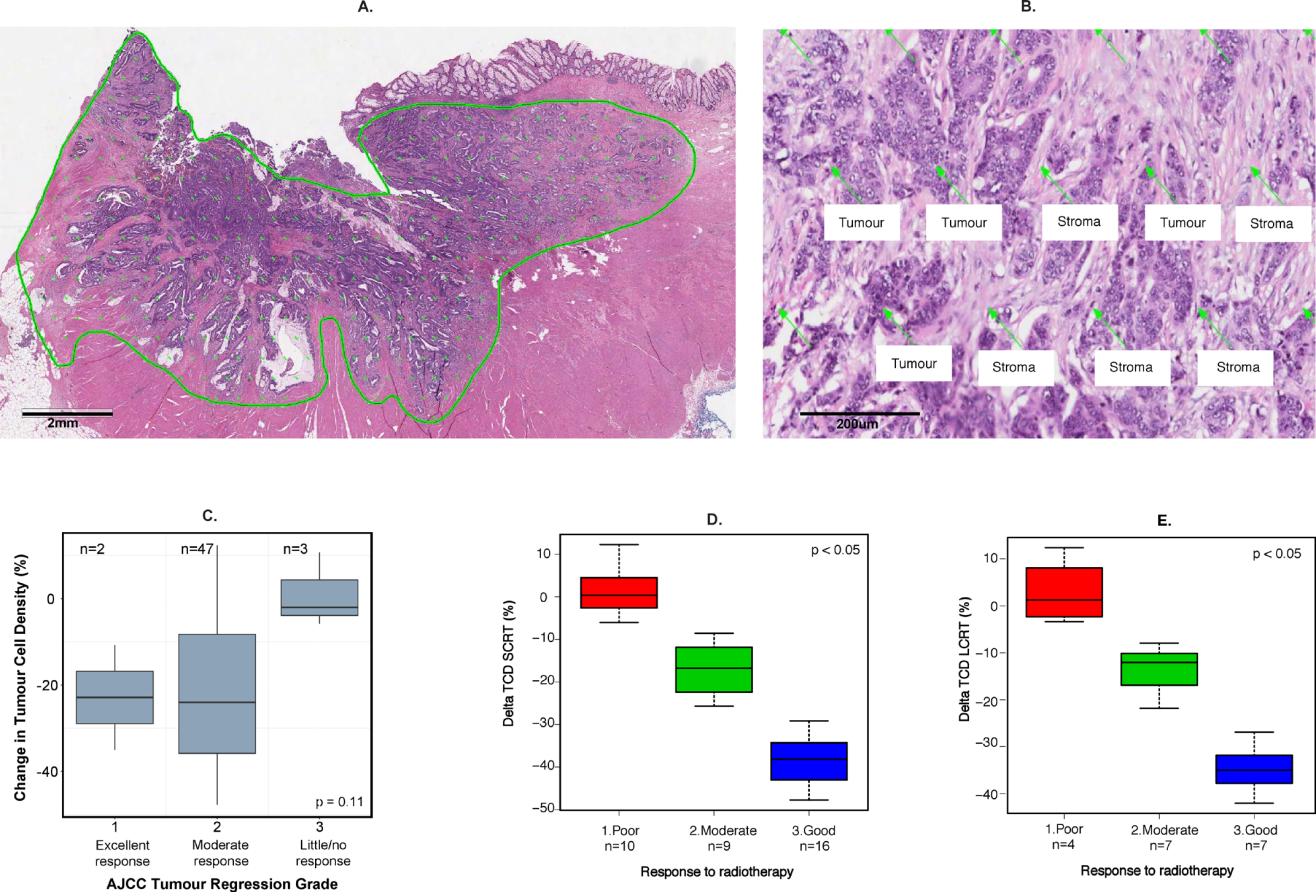
Methodology for evaluation of tumor cell density (TCD) and allocation of radiotherapy response categories. (A) Virtual graticule of approximately 300 points applied to tumor area on a digital H&E section. (B) Demonstration of TCD point scoring. (C) Box and whisker plot to show the relationship between ΔTCD and American Joint Committee on Cancer (AJCC) Tumour Regression Grade System (TRG). (D, E) Allocation of three radiotherapy response groups according to change in TCD (ΔTCD) for short-course radiotherapy (SCRT) (D) and long-course chemoradiotherapy (LCRT) (E). The Kruskall-Wallis test was used for statistical comparisons.

All cases were reviewed and TRG was evaluated according to AJCC TNM Staging Manual (8th Edition)^15^ in resection specimens by a specialist gastrointestinal pathologist. In order to overcome the subjectivity inherent in the TRG system, a previously described quantitative method (TCD) was also evaluated.^8 9^ In brief, TCD evaluation involves quantification of 300±15 individual points distributed across the whole tumor area on digital H&E-stained tumor sections (figure 1A, B). The selection of 300 points is derived from earlier modeling studies which identified that at least 250 points were needed to accurately quantify the percentage of tumor cells. Baseline and post-treatment TCD were assessed by an independent observer, blinded to TRG evaluation.

### Gene expression profiling

Following demarcation of tumors on H&E slides by a specialist gastrointestinal pathologist, tumor areas were macrodissected from unstained slides to capture the whole tumor microenvironment. Nucleic acids were extracted using The RecoverAll Total Nucleic Acid Isolation Kit and quantified using Qubit Fluorometry (both from Thermo Fisher Scientific, UK), according to manufacturer’s instructions. The expression of 760 genes included in the NanoString panCancer Immune Panel (NanoString Technologies, Seattle, Washington) were measured according to manufacturer’s instructions and normalized using positive and negative controls and the housekeeping genes included in the panel. Only genes with non-zero expression in 75% or more of the samples were retained.^16^ The presence of a batch effect in log_2_ transformed and normalized data was assessed using exploBatch^17^ and corrected using *ComBat* from SVA Bioconductor-based R package.^18^

### TCD analysis

**Figure 2:**
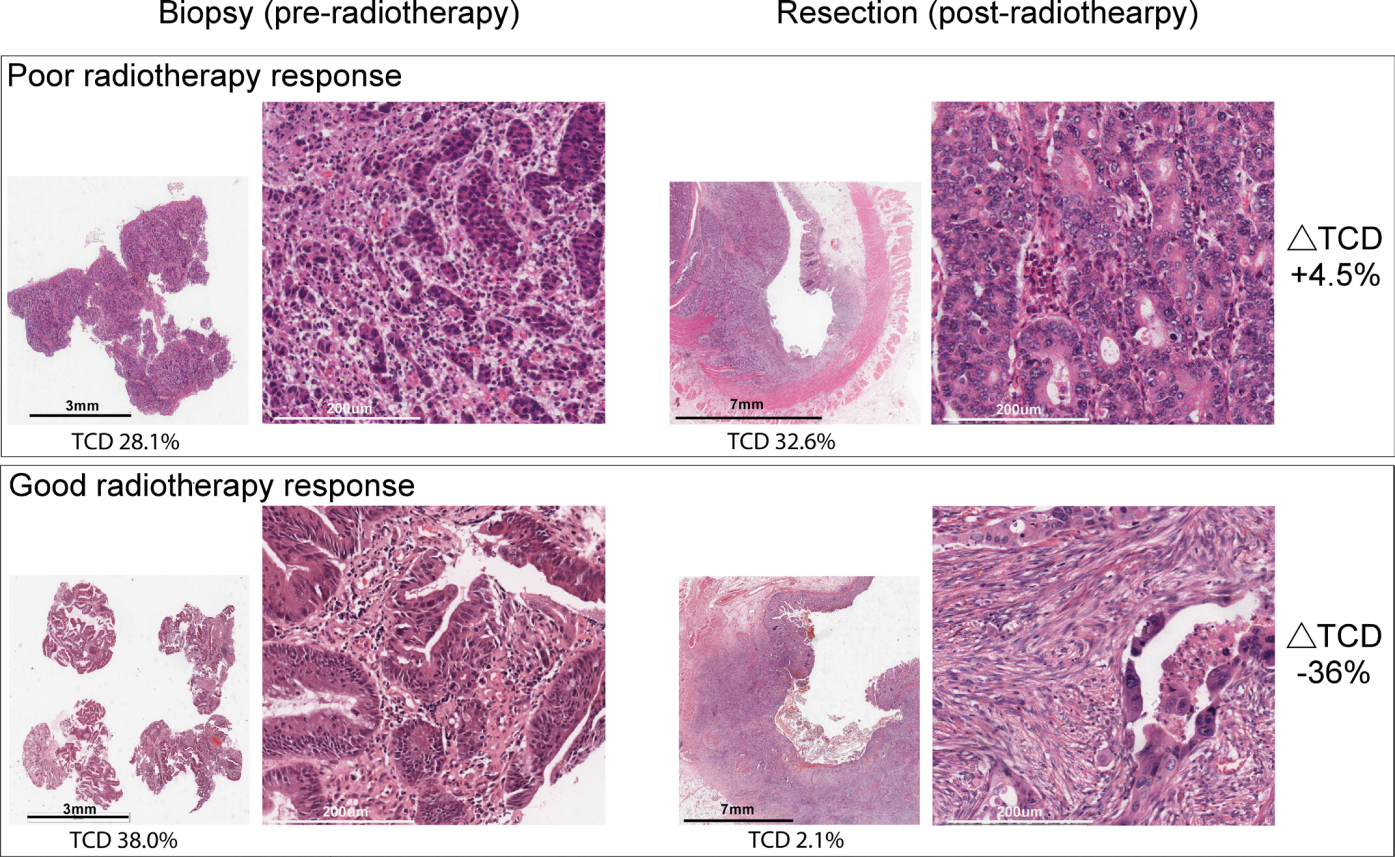
Example of H&E staining preradiotherapy and postradiotherapy in good versus poorly responding tumors defined using the change in tumor cell density (ΔTCD).

Patients’ characteristics were summarized using descriptive statistics. The difference between resection specimen and baseline TCD (ΔTCD) was calculated; its association with TRG was assessed using the Kruskal-Wallis test. Next, ΔTCD was used to classify each patient’s tumor response into three categories (good, intermediate or poor) using k-means clustering. To determine GEP that were most relevant to differential radiotherapy responses, we excluded the intermediate response group and compared good versus poor responders in the majority of subsequent analyses (an H&E demonstration of good vs poor responders is shown in figure 2).

### Immune gene expression, pathways and cell type analysis

The Estimation of STromal and Immune cells in MAlignant Tumours using Expression data (ESTIMATE) algorithm^19^ was used to infer tumor purity and the fraction of stromal and immune cells in baseline biopsies of tumors with good versus poor radiotherapy response. Following this, significance analysis of microarrays (SAM) was used to identify individual genes with a significant difference in expression at baseline between good and poor responders.^20^ A comparison of intermediate responders’ GEP with those of the good and poor responders was also performed. Following this, functional annotation of the significant genes according to pathways (*hypeR R* package^21^) and cell types was performed (using an Enrichr-based gene enrichment analysis^22^), and two publicly available gene sets (ARCHS4 and the Human Gene Atlas).^23 24^ ARCHS4 is a published database of cell types derived by mining RNAseq data from public gene expression databases.^23^ For Human Gene Atlas, the authors created custom microarrays to measure gene expression of known and predicted gene proteins associated with tissues and cell types.^24^ We used Enrichr combined score analysis to perform enrichment of cell types in our radiotherapy-treated rectal gene expression cohort.^22^ The pathways to genes plots were done using multiple packages—*clusterProfiler*,^25^ *msigdbr*^26^ and *enrichplot*.^27^

Longitudinal (treatment-induced) significant changes in gene expression were evaluated in paired samples of good and poor responders in two separate SAM analyses.^20^ For pathway analysis, the Molecular Signature Database (MSigDB)’s hallmarks gene sets^26^ and *hypeR R* package^21^ was used to describe the biological pathways represented by differentially expressed genes. Similarly, longitudinal changes in the 18-gene T-cell-inflamed GEP,^28^ that is associated with prediction of immunotherapy response, were evaluated in good and poor responders. Of note, our panel included 16 of the 18 genes in the GEP. Finally, longitudinal immune cell type changes in good and poor responders were inferred using single-sample gene set enrichment analysis^29^ and immune enrichment signatures (IES) from Rooney *et al*.^30^

### Validation datasets and analysis

The use of different GEP platforms, non-overlapping genes and inconsistent measures of radiotherapy response across published reports poses challenges for independent validation of findings. To address this, meta-analysis at the level of gene sets/pathways was used to compare upregulated pathways according to response in our datasets with those in two independent cohorts.^31 32^ In addition, as our study cohort and the above two cohorts did not have survival outcomes available, the impact of differential gene expression on disease-specific survival (n=182) was assessed in a third independent and publicly available cohort—GSE87211 (n=188), where patients were treated with CRT.^33^

The first validation cohort consisted of GEP from 15 patients receiving preoperative radiotherapy. Radiotherapy response was defined using the Mandard tumor regression grade system where TRG1 and TRG2 were considered to be responders and TRG3, TRG4 and TRG5 were classified as non-responders. PrimeView Affymetrix arrays were used for GEP using RNA extracted from microdissected epithelial and stromal components of fresh frozen preradiotherapy tumor tissue biopsies.^31^ We used the pathway analyses data, from a supplementary table of the Goncalves-Ribeiro study, derived from dysregulated genes in the stromal component of radiotherapy non-responders (compared with responders).^31^

The second validation cohort consisted of 23 patients with rectal cancer treated with preoperative radiotherapy to a dose of 45 Gy in 25 fractions, 5 days per week for 5 weeks, ± 5.4 Gy boost. The AJCC TRG system was used to define radiotherapy response in surgical specimens; tumors were classified as “total responders” when assigned to TRG0, “partial responders” when TRG1 and TRG2, and “non-responders” when TRG3. Proteomics analysis was performed using high performance liquid chromatography separation, coupled to mass spectrometry by the authors. We used the list of 139 proteins (from the supplementary table of the original publication) that discriminated non-responder from total responders to preoperative radiotherapy from Chauvin *et al*.^32^ We applied enrichment analysis using “Investigate Gene Sets” from the MSigDB^26^ to discern the changes in pathways associated with the “hallmarks gene sets”.

The third validation cohort consisted of gene expression data (n=188) and disease-specific survival outcomes of 182 patients with locally advanced rectal cancer treated with preoperative radiotherapy to a dose of 50.4 Gy.^33^ GEP was carried out by the authors using the Human 4×44 K v2 array platform from Agilent Technologies (G4845A). Disease-specific survival measured from the time of surgery to death or last clinical follow-up was used.

To classify the samples from the third cohort into two classes that resemble poor and good responders from our cohort, average (mean) expression of 40 genes, significantly upregulated in poor responding samples from our cohort, was calculated for each sample. The optimal cutpoint for the mean expression was further calculated using the maximally selected rank statistics from “survminer” R package using “surv_cutpoint” command. The optimal cutpoint provided two classes with high (representing poor-like responders) and low (representing good-like responders) expressors. These expressor groups arising from poor responding genes were further used to perform disease-specific survival and further gene expression analysis.

### Immunohistochemistry

In order to provide further protein-level confirmation of the above gene expression findings, dual staining of CD68 and CD163 using immunohistochemistry was carried out in a limited number of samples due to the constraints of tissue availability. The immunohistochemistry methods for this are described in the online supplemental methods.

10.1136/jitc-2020-001717.supp1 Supplementary data

### Statistics

Kruskal-Wallis, Fisher exact and t tests were applied where necessary. Survival analysis was carried out using Kaplan-Meier methods and the log rank test.

## Results

### Study population and ΔTCD-based response to RT/CRT

**Figure 3:**
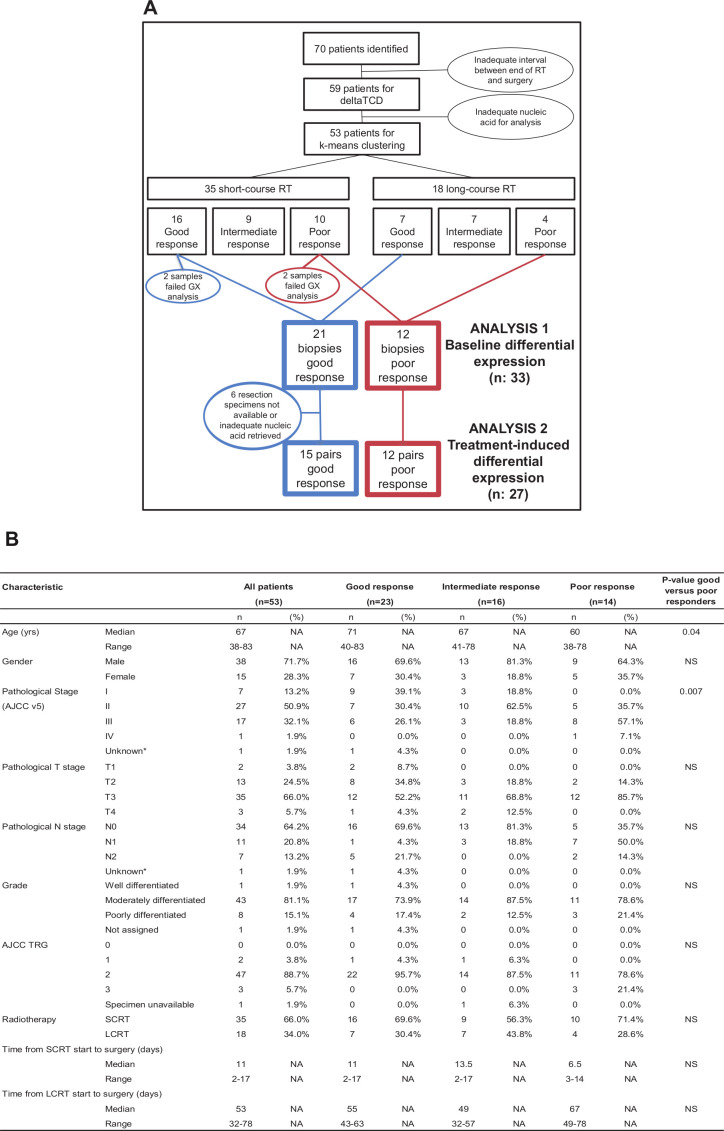
Consolidated Standards of Reporting Trials (CONSORT) diagram and clinical characteristics of samples. (A) CONSORT diagram to show patient samples’ flow through the study. (B) A table with baseline characteristics of patients with valid gene expression data. Good, intermediate and poor response categories are defined by ΔTCD. The Kruskall-Wallis test was used for statistical comparisons. AJCC, American Joint Committee on Cancer; LCRT, long-course chemoradiotherapy; SCRT, short-course radiotherapy; TRG, tumor regression grade; TCD, tumor cell density; NA, Not available; NS, not significant.

A total of 140 archival blocks from 70 patients were identified (figure 3A). Eleven patients with a prolonged interval between the end of radiotherapy and surgery were excluded. Fifty-three cases with successful RNA extraction from diagnostic biopsies were available for analysis, of whom 52 cases had TRG scores. Patients’ characteristics are tabulated in figure 3B. In line with the published literature,^3 4^ the median age at diagnosis was 67 years and 72% of the patients were male. At least 45/53 (85%) of tumors were staged greater than pathological stage I after neoadjuvant treatment.

TRG 2 (moderate response) was ascribed to 90.4% (47/52; leaving out one sample with TRG status unavailable) of the tumors; only 2 and 3 patients were scored as TRG 1 and 3, respectively, and no patients had a pathCR (TRG 0). Hence, the TRG, consistent with its known limitations,^3^ was not considered a suitable endpoint to identify differential response to neoadjuvant treatments. Conversely, the ΔTCD scores ranged from 12.3% to −47.7% and from 12.4% to −42.1% in patients treated with SCRT and LCRT, respectively.

Despite very few patients being categorized as TRG 1 or 3, a marginal association between TRG and ΔTCD was seen (figure 1C). The three patients with TRG 3 had minimal ΔTCD (mean ΔTCD 0.96%) whereas the two patients with TRG 1 had much more substantial ΔTCD (mean ΔTCD −22.8%). As ΔTCD scoring better represented heterogeneous responses to neoadjuvant therapy, ΔTCD was subsequently used to define response categories. Following k-means clustering into three response groups, 23 good, 16 intermediate and 14 poor responders were identified (figure 1D, E). An example of H&E staining preradiotherapy and postradiotherapy in good versus poorly responding tumors is shown in figure 2. Here, the poorly responding postradiotherapy resection specimen shows a high density of residual tumor cells. Conversely, in the resection specimen showing a good response, there are very few tumor cells and the tissue is predominantly replaced by reactive stroma.

A comparison of clinicopathological characteristics in good versus poor responders indicated that poor responders were significantly younger with higher pathological stage. Importantly, there was no significant difference between the two response categories and type of neoadjuvant treatment (SCRT or LCRT) or the interval between radiation and surgery (figure 3B). This reinforces the biological meaning of ΔTCD and suggests that significant associations with variation in radiotherapy technique are unlikely, at least in this cohort.

### Immune profiles of baseline diagnostic biopsies in patients with ΔTCD good versus poor response to radiotherapy

**Figure 4:**
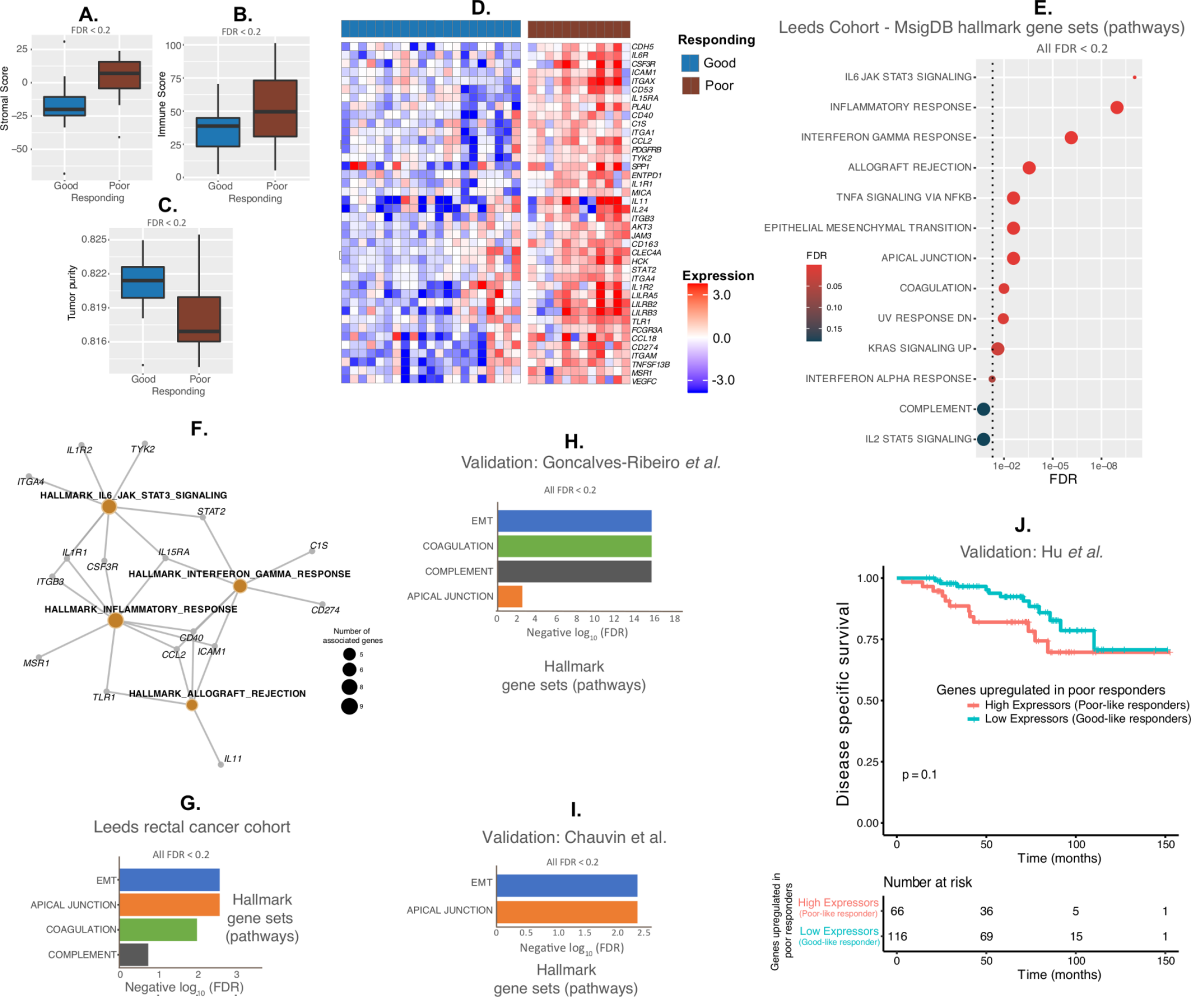
Analysis of baseline gene expression and pathways in preradiotherapy diagnostic biopsies. (A–C) Stromal score (A), immune score (B) and tumor purity (C) in baseline biopsies of tumors with good versus poor radiotherapy response using the Estimation of STromal and Immune cells in MAlignant Tumours using Expression data (ESTIMATE) algorithm.^19^ (D) Heat map of 40 genes showing significantly higher expression in the biopsies of poorly responding tumors versus tumors with a good response. (E) Pathway analysis of upregulated pathways in poor responders using Molecular Signature Database (MSigDB) Hallmarks. (F) Network showing upregulated pathways and corresponding genes in poorly responding tumors at baseline. (G–I) Pathway analysis in Leeds cohort (using hypergeometric test from hyper^21^ (G), validation cohort pathway analysis of gene signatures from Goncalves-Ribeiro *et al* (FDR values for pathways from the authors)^31^ (H) and validation cohort pathway analysis of protein signatures from Chauvin *et al*^32^ using GenePattern’s Investigate Gene Sets hypergeometric test^26^ (I). (J) Kaplan-Meier curves to show disease-specific survival outcomes of samples, from a publicly available gene expression (GSE87211) dataset treated with chemoradiotherapy, according to high or low expression of the 40 genes highly expressed in poor responding samples shown in (D). Expressors refer to samples with high or low expression of 40 genes from (D). The definition of the groups was described in methods sections. Since the 40 genes are highly expressed in poor responding samples in (D), high expressors were named as poor-like and low expressors as good-like samples from GSE87211 samples. The log-rank statistical test was applied for p value significance.

Use of the ESTIMATE algorithm, which infers cancer versus stromal/immune content at the gene expression level, showed that, at baseline, poorly responding tumors contained a significantly higher fraction of stromal and immune cells (ie, lower tumor purity) than tumors with a good response (figure 4A–C). When the immune GEP at baseline of good and poor responders (n=33) were compared using SAM analysis, 40 genes showed significantly higher expression in poor compared with good responders (figure 4D). These included genes involved in immune checkpoint inhibition (*CD274* or *PD-L1*), immune regulation (*IL11*, *IL15RA*, *IL6R*) and integrin signaling (*ITGA1*, *ITGA4*) as well as markers of macrophages (*CD163*, *ITGAM*) and stromal fibroblasts (*PDGFR*-β). Eight genes showed significantly (FDR≤0.05) differential expression when the three responder groups—good, intermediate and poor were compared by SAM analysis. Of these 8 genes, 7 genes overlapped with the 40 poor responder genes suggesting that the intermediate responders were of an immune phenotype in between the good versus poor responders (online supplemental table 1).

Pathway analysis using the 40 upregulated genes in poorly responding tumors revealed 13 significantly (FDR<0.2) upregulated hallmark pathways, where more than 50% of the pathways were associated with direct immune regulation (figure 4E). The genes associated with four pathways showing the most significant upregulation in our cohort (IL6/JAK/STAT3 signaling, inflammatory response, interferon gamma response and allograft rejection) are shown as a pathways network with associated genes in figure 4F. More than 50% of these genes were shared by the four pathways representing similar mechanism(s) of antiviral-like immune regulation operating in the pretreated poor responding samples compared with good responding samples.

We used two independent cohorts of rectal cancer samples for validation using pathway analysis derived using gene and protein expression, respectively. A third independent cohort with disease-specific survival was used for validation. Upregulation of the epithelial–mesenchymal transition, and apical junction pathways were validated in both independent cohorts and pathways of complement and coagulation were validated in one independent cohort (figure 4G–I, online supplemental table 2). These two cohorts served as independent validation using both gene and protein expression profiles. The pathways from the first cohort were generated from microdissected stroma. Hence, the genes enriched in the poor responding samples are potentially from stroma. In the third validation cohort, there was a trend towards inferior disease-specific survival outcomes in patients (high expressors or poor like) whose tumors had increased expression of the 40 genes upregulated in poor responders (from our training cohort) compared with those patients with tumors not showing increased expression (low expressors or good like; figure 4J). The mean expression of the 40 upregulated genes in poorly responding tumors in high versus low expressors and their expression patterns as a heatmap in the third validation cohort are shown in online supplemental figure 1. These results show pathway-based and survival-based validation of the responder groups.

10.1136/jitc-2020-001717.supp2 Supplementary data

10.1136/jitc-2020-001717.supp3 Supplementary data

**Figure 5:**
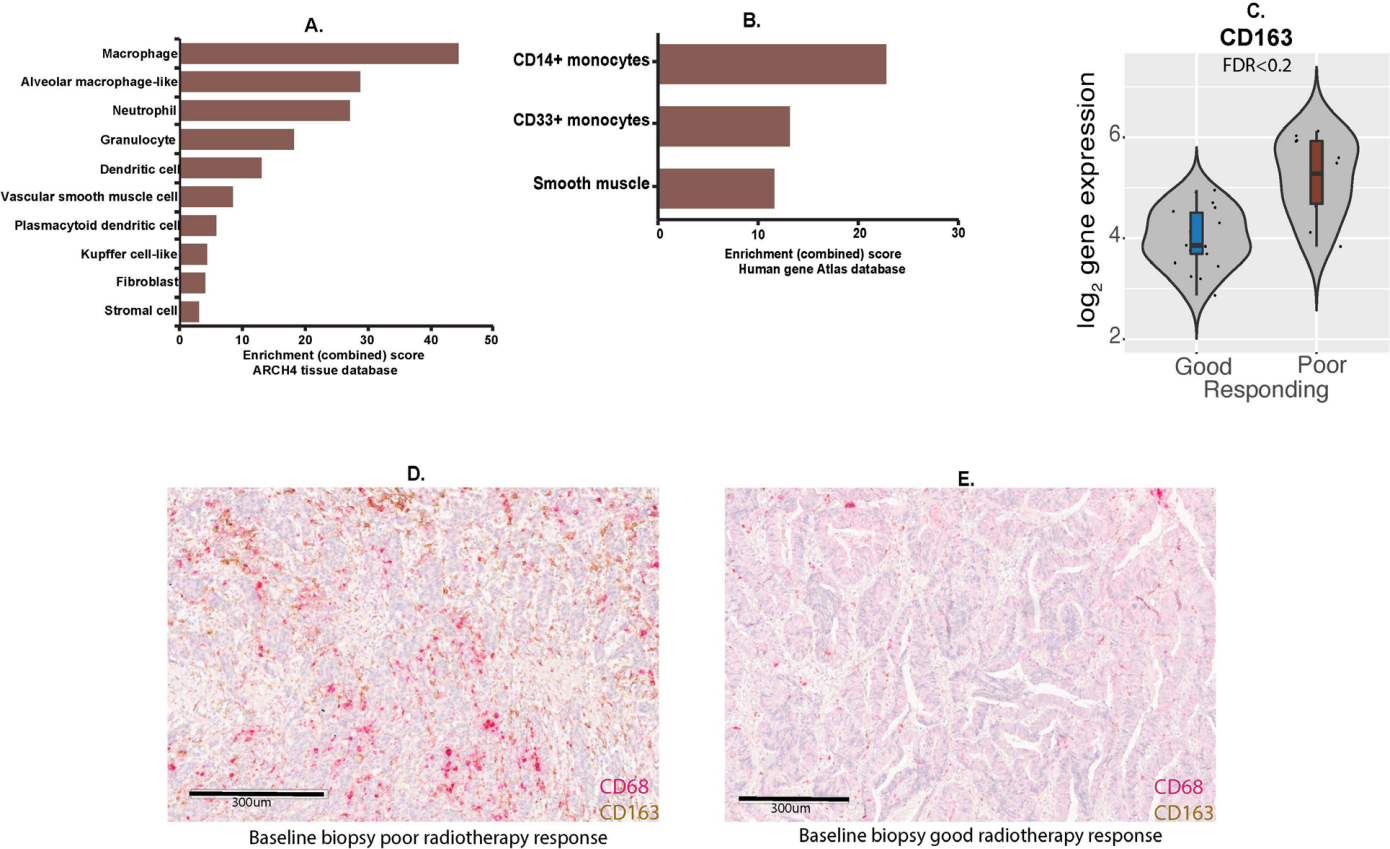
Analysis of baseline cell populations in preradiotherapy diagnostic biopsies. (A, B) Enrichment analyses using ARCHS4 (A)^23^ and human gene atlas (B)^24^ tissue databases showing increased myeloid/monocyte cell populations and stroma in poorly responding tumors versus tumors with a good response. (C) Expression of *CD163* gene in good (n=21) versus poor (n=12) responding tumors in baseline biopsies. (D, E) Immunohistochemistry for CD68 and CD163 protein in baseline biopsies of tumors showing poor (D) versus good (E) radiotherapy response, respectively.

Functional annotation of the 40 genes using publicly available resources and enrichment analysis demonstrated their significant association with macrophages, monocytes and dendritic cells, as well as stromal cells including fibroblasts and smooth muscle cells (figure 5A, B, online supplemental tables 3–6). CD163 is marker of alternatively-activated (M2-like) macrophages which are thought to have tumor-promoting effects.^34^ *CD163* gene was significantly upregulated in poorly responding tumors (figure 5C). Dual staining of CD68 and CD163 using immunohistochemistry was possible for a very limited number of biopsies due to tissue availability. Nevertheless, figure 5D, E demonstrate marked upregulation of both CD68 and CD163 staining in the baseline biopsy of a poorly responding tumor when compared with the baseline biopsy of the tumor showing a good radiotherapy response.

10.1136/jitc-2020-001717.supp4 Supplementary data

10.1136/jitc-2020-001717.supp5 Supplementary data

### Radiation-induced immune changes in good and poor responders

**Figure 6:**
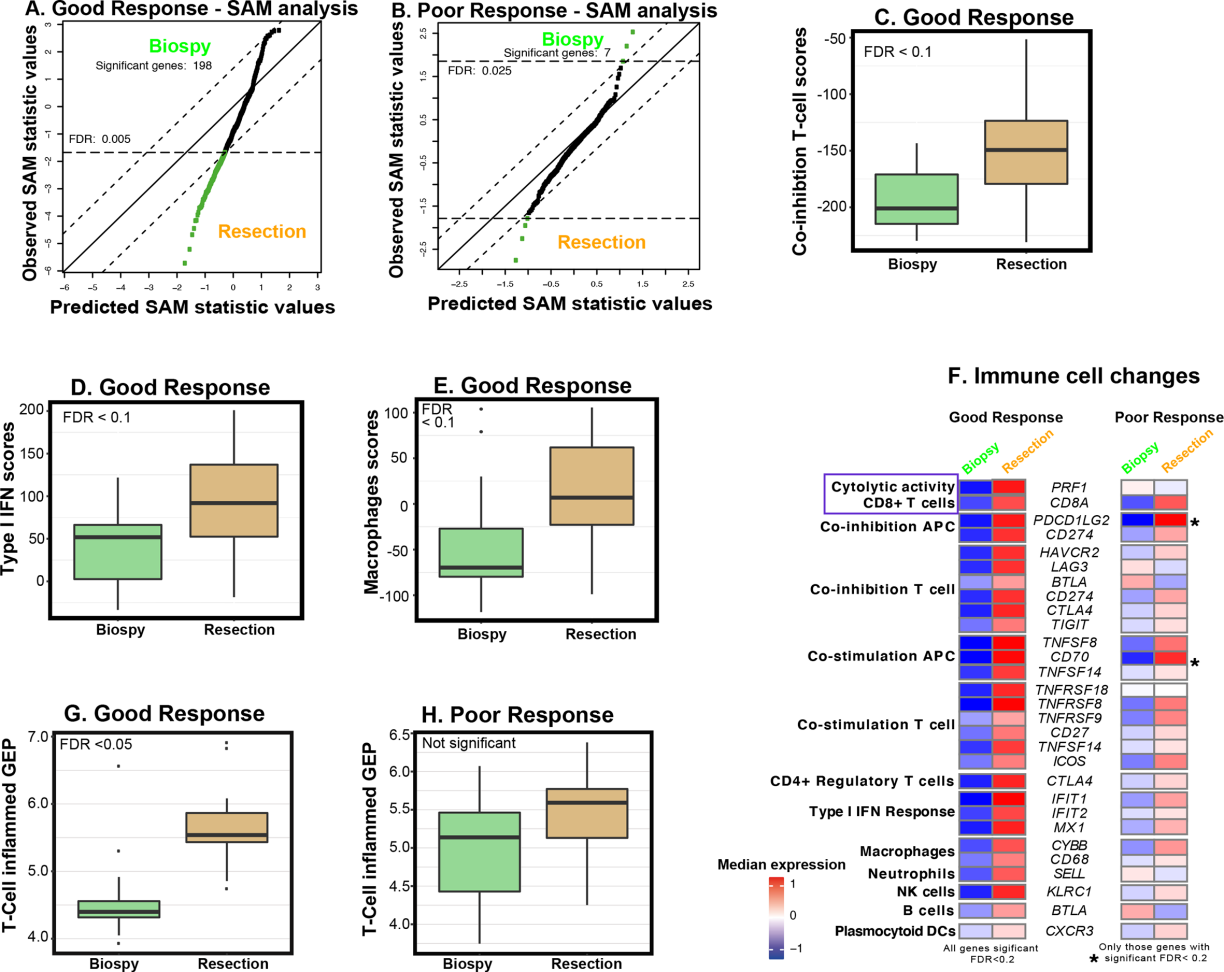
Longitudinal analysis of changes in immune gene expression in matched preradiotherapy diagnostic biopsy and postradiotherapy resection specimen. (A, B) Significance analysis of microarrays (SAM) plot to show 198 genes with a significant difference in expression between pre-RT biopsy and post-RT resection in tumors with a good response (A) and those with a poor response (B). Four upregulated genes during RT in (B) include *C7, CHIT1, CXCL12* and *SPP1* while downregulated genes during RT include *CCL28, DMBT1* and *CEACAM1*. Significant genes are shown in green while non-significant genes are shown in black. (C–E) Box plots showing changes in immune enrichments scores (IES)^30^ for T cell coinhibition (C), type I interferon (IFN) (D) and macrophages (E) between pre-RT biopsies and post-RT resection specimens in tumors showing a good RT response. (F) Heatmaps to show changes in significant IES in pre-RT biopsies and post-RT resection specimens in tumors showing a good RT response and a poor RT response. Certain genes were repeated depending on their immune cell type/regulation categories. A blue box highlights increased cytolytic activity and CD8+ T cells in post-RT resection samples compared with pre-RT biopsy samples. (G, H) Change in the T-cell-inflamed GEP^28^ between pre-RT biopsy and post-RT resection in good responders (G) and poor responders (H).

Significant longitudinal changes in immune gene expression during radiotherapy were evaluated with two separate SAM analyses in 15 paired samples (pretreatment biopsies and resection specimens) from patients with a good response and in 12 paired samples from poor responders. In good responders, 198 immune genes showed significant upregulation (figure 6A and online supplemental figure 2) in the postradiotherapy resection specimen when compared with the preradiotherapy biopsy. However, in poor responders, the expression of only seven genes was significantly modified by radiotherapy treatment (*C7, CHIT1, CXCL12*, and *SPP1* were upregulated and *CCL28, DMBT1* and *CEACAM1* were downregulated; figure 6B).

10.1136/jitc-2020-001717.supp6 Supplementary data

Significant increases in coinhibition of T-cells, type I interferon response and macrophages were demonstrated in good responders (figure 6C–E, the genes used in these signatures are show in figure 6F). Conversely, no significant changes were observed in poor responders which, as described earlier, appeared to have a higher macrophage score at baseline (online supplemental figure 3A-C). Similarly, a significant increase in the T-cell-inflamed GEP signature was demonstrated in good responders, but not in poorly responding tumors, which appeared to have a more inflamed GEP signature at baseline than good responders (figures 6G and 4D). Evaluation of IES showed a consistent, significant increase in the expression of 25 (out of 56) genes associated with multiple immune cell types (including CD8+ T cells and perforin; *PRF1*, and macrophages; Figure 6F) and processes in good responders. Conversely, such immune activation was not present in poor responders (except for two significant genes—*PDCD11LG2* and *CD70*; figure 6F), which showed increased macrophage score at baseline (figure 5C).

10.1136/jitc-2020-001717.supp7 Supplementary data

**Figure 7:**
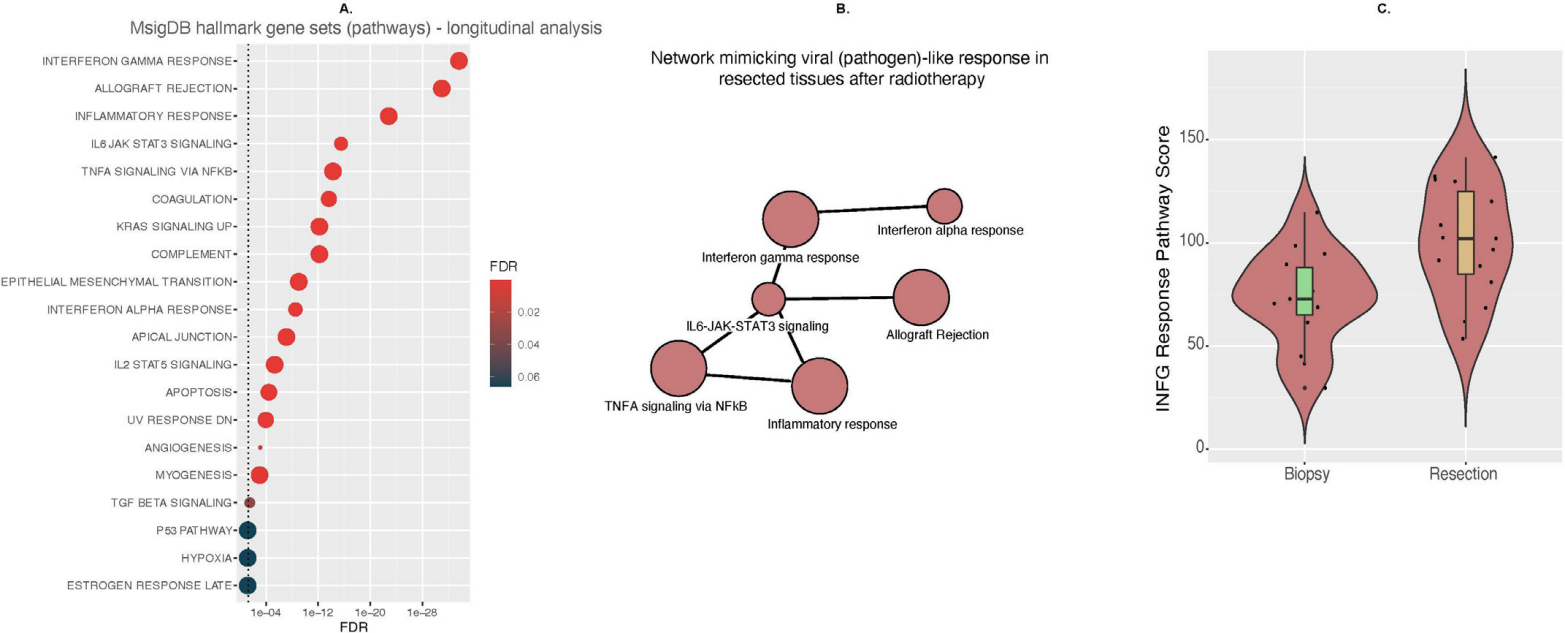
Pathway analysis of longitudinal gene expression changes during radiotherapy in tumors with a good response. (A) Pathway analysis of upregulated pathways during radiotherapy in good responders using Molecular Signature Database (MSigDB) and hallmarks gene sets. (B) Network mimicking viral (pathogen)-like response in resected tissues after radiotherapy. (C) Change in interferon-gamma response pathway score during radiotherapy in tumors showing a good response. IL, interleukin; IFN, interferon.

To understand the biological pathways represented by the 198 upregulated genes in tumors with a good response, pathway analysis was performed. A highly significant upregulation of interferon gamma response, allograft rejection and inflammatory response pathways was seen alongside several other significantly (FDR<0.2) upregulated pathways (figure 7A). Taken together, these suggest an immunostimulatory response to radiotherapy, together with radiation-induced replacement of tumor cells with reactive stroma. Figure 7B shows the six most significantly upregulated pathways and their associated genes in a network with potential activation of antiviral pathways. The downstream effect of this activation is an interferon gamma response. We observed a highly significant increase in interferon-gamma response pathways scores in good responders (figure 7C).

## Discussion

In this study, immune GEP was conducted in 53 paired preradiotherapy and postradiotherapy rectal cancers to identify immune genes, pathways and cells associated with radiotherapy response. A unique quantitative method (*ΔTCD*) identified immune-related features of rectal cancers that showed a differential response to neoadjuvant RT/CRT. Although 47 of 52 tumors (90.4%) presented with the same TRG, *ΔTCD* allowed further refinement of response assessment with identification of three distinct groups and further comparison of good versus poor response groups. Of note, our study cohort did not include patients with a pathCR to radiotherapy.

The 40 immune genes and associated pathways significantly upregulated in the baseline biopsies of tumors showing a poor response may potentially represent mechanisms of resistance to RT/CRT. These genes are a reflection of an inflamed yet immunosuppressive microenvironment enriched for specific myeloid populations and fibroblasts (stroma enriched). In addition, the upregulation of integrins suggests distinct interactions between the extracellular matrix and cells within a fibrotic (*PDGFR-β*) and proangiogenic (*VEGFC*) tumor microenvironment. The corresponding upregulated biological pathways include epithelial–mesenchymal transition, apical junction signaling, allograft rejection and complement activation, three of these were validated in independent cohorts. Furthermore, the enrichment of the 40 genes in a separate cohort showed poor prognosis, demonstrating an adverse effect associated with the poor response to RT/CRT.

These data support further preclinical and clinical evaluation of agents, before and/or in combination with RT/CRT, which may modify these immunosuppressed and radio-resistant phenotypes and increase the chance of a good pathological response, which is associated with better long-term outcomes. We showed that poor radiotherapy responses were more common in younger patients; early onset sporadic rectal cancer appears to be increasing and there is a particular need for more biological understanding and better therapeutic strategies in this context. A number of early phase clinical trials testing the feasibility and potential activity of immunotherapy or other drugs targeting the microenvironment in conjunction with RT/CRT are currently recruiting patients,^35–38^ and our study supports the biological rationale of testing immune checkpoint inhibitors in particular in this setting. The heterogeneous immune phenotype at baseline demonstrated in the current study may encourage and inform the use of biomarker-selected approaches in future.

In this study, baseline gene expression in small diagnostic biopsies was successfully measured using the nCounter platform (NanoString Technologies). This technology is approved for clinical use to help assess the risk of recurrence and potential benefit from adjuvant chemotherapy in breast cancer.^39^ Recently, our group validated customized small gene panels for subtype prediction using a protocol modified for potentially affordable cost if applied in the clinic.^16 40^ Hence, these studies support the feasibility of using small customized gene expression signatures for prospective patient selection.

The longitudinal analyses have elucidated distinct differences in the radiation-induced immune response between good and poorly responding tumors which, to our knowledge, have not been described before. Tumors with a good response showed both increased T-cell inflammation and increased immune cytolytic activity, suggestive of a transition from immune “cold” to a more immunologically “hot” phenotype after RT/CRT. In this transition, the increase in the cytolytic marker perforin-1 (*PRF1*) at the gene expression level is accompanied by a potential immunoregulatory response involving upregulation of immune checkpoints including *CD274*/*PD-L1, LAG3*, *CTLA4, BTLA, TIGIT* and others (figure 6F), as described previously.^30^ A timely identification of this immunoregulatory response after RT/CRT may provide a therapeutic opportunity to further enhance tumor response by the additional adjuvant use of immune checkpoint inhibitors.

In contrast, tumors with a poor response showed upregulation of IL6/JAK/STAT3, inflammatory and interferon pathways, among others, at baseline, with much less of a change in immune phenotype during neoadjuvant treatment. These pathways closely overlap those upregulated following radiotherapy in good responders; however, the context in which this upregulation occurs is very different. At baseline, tumors with a poor response show *intrinsic* features of chronic inflammation and chronic interferon signaling, together with stromal fibroblast and myeloid cell enrichment. In both preclinical and clinical studies, such tumors are associated with immunosuppression and inferior outcomes following radiotherapy.^41 42^ Reciprocal oncogenic signaling between cancer and stromal cells and increased extracellular matrix may also contribute to poor therapy responses.^43 44^ In contrast, the longitudinal acute upregulation of inflammatory and interferon pathways in good responders is an *acquired* phenotype induced by therapy and associated with a reactive “wound repair” stroma.

Collectively, the above findings demonstrate the need to understand the baseline immune context of tumors alongside longitudinal treatment-induced immune changes, and highlights the complexity underlying the biology of tumor ‘inflammation’, with regard to baseline tumor intrinsic and treatment-induced characteristics, and their relevance to clinical outcome. For example, in poor responders, immune checkpoint inhibitors may be more powerful if used before/during RT/CRT as an “immune primer” to revert the baseline immunosuppressive tumor phenotype with high levels of *PD-L1* expression. In our study, *SPP1* was one of the genes that was both upregulated in baseline biopsies of poor responders and showed a significant longitudinal increase in the same poor responders. A recent single cell sequencing analysis of immune and stromal populations in 18 colorectal tumors identified a proangiogenic, CAF-enriched, tumor-promoting SPP1+ tumor-associated macrophage population (TAM).^45^ It is possible that our study identified *SPP1* expression on tumor-promoting TAM which drive therapy resistance, in which case specific targeting of this population during rectal cancer radiotherapy is worthy of further study.

A longitudinal increase in the expression of genes associated with a type I interferon response was seen in tumors showing a good RT/CRT response. These data lend support to preclinical observations that the cGAS/STING/type 1 interferon pathway, the biology of which is critical in the immune response to viral infection, is important in the radiation-induced immune response.^46 47^ They also indicate that further study of the relationship between radiation-induced DNA damage and the immune response is warranted, including possible synergy with DNA damage response inhibitors^13 48^

Several other studies investigating the role of different immune cell markers have been reported in rectal cancer.^13 14 49 50^ In preradiotherapy biopsies, increased CD8+ T cells have been associated with a complete response to radiation,^13^ and high infiltration of both CD3+ and CD8+ cells with increased tumor downstaging after preoperative chemoradiotherapy.^49^ A study characterizing CD8+ and FOXP3+ tumor-infiltrating lymphocytes in 237 biopsy and resection pairs showed that a significant decline in the intratumoral CD8+/FOXP3+ ratio after radiotherapy was associated with superior survival outcomes.^50^ However, this series looked at only CD8 and FOXP3 staining, and the relationship between these markers and more global gene expression reflecting the activation/suppression of all immune cell subtypes, remains unknown. Nevertheless, the above studies and our work collectively suggest that both tumor immune cell phenotyping and immune GEP may have roles in guiding personalized and/or combination radiation treatment.

This study has a number of limitations. First, our study cohort is fairly small, which means our findings are primarily hypothesis generating and precludes separate evaluation of LCRT and SCRT schedules. Nevertheless, there are not many studies in rectal cancer RT/CRT that have used a greater number of samples than our study. In order to further validate our findings, we have used two different cohorts (n=38) and pathway analysis derived from gene and protein expression, respectively. Second, long-term survival outcomes were also not available in our study cohort. However, in a third independent cohort (n=182), we have shown a trend towards inferior disease-specific survival in patients with the same upregulated genes as poor responding tumors in our study, despite the fact that disease-specific survival will be impacted by variation in surgical technique, systemic treatment following tumor recurrence and other factors that are unrelated to radiotherapy response. Furthermore, the TCD, used as an endpoint to define response, was previously significantly associated with recurrence-free survival in colorectal cancer, independently of age, pT-stage, pN-stage and extra-mural venous invasion.^8^ Additionally, in a recent phase II study of neo-adjuvant chemotherapy and radiotherapy, TRG and TCD showed a highly significant association and, despite the small study size, a borderline significant relationship between TCD and progression-free survival was seen.^51^

We also acknowledge the possible presence of sampling errors associated with biopsies and, consequently their gene expression; validation of these results in more contemporary studies with TCD analysis, ideally with multiple biopsies taken from the same tumor to account for intratumoral heterogeneity, would be worthwhile. Finally, we acknowledge that the addition of immune checkpoint inhibition may lead to paradoxical T cell exhaustion. Further study is needed to confirm the validity of specifically-timed radiotherapy/immunotherapy combinations suggested in this study, particularly in view of the lack of efficacy of immune checkpoint inhibition in microsatellite stable colorectal cancer.

In summary, this study has used a unique quantitative evaluation (Δ*TCD*) to elucidate differences in RT/CRT response; Δ*TCD* has the potential to enhance treatment stratification in the clinic. Second, we have demonstrated the immunostimulatory role of RT/CRT in a subgroup of patients whose tumors have a baseline immunologically cold phenotype and continue to show a good response to RT/CRT. Third, we have performed pathway analysis to show potential viral mimicry phenotype hijacked by good responding tumors after radiotherapy to evade immune surveillance. Finally, this study suggests potential new therapeutic strategies using immunomodulatory agents and other targeted drugs in combination with RT/CRT to modify radiorefractory inflamed tumor phenotypes. Precisely timed administration of such agents in selected groups of patients may enable improved responses to neoadjuvant therapies in rectal cancer.

## Data Availability

Data are available upon reasonable request. The data will be deposited in an appropriate repository.
