# Correction: *Differential and longitudinal immune gene patterns associated with reprogrammed microenvironment and viral mimicry in response to neoadjuvant radiotherapy in rectal cancer*

**DOI:** 10.1136/jitc-2020-001717corr1

**Published:** 2021-06-16

Wilkins A, Fontana E, Nyamundanda G, *et al*. Differential and longitudinal immune gene patterns associated with reprogrammed microenvironment and viral mimicry in response to neoadjuvant radiotherapy in rectal cancer. *J Immunother Cancer* 2021;9:e001717. doi: 10.1136/jitc-2020-001717

This paper has been updated since first published to amend author and ethics committee details.

