# Supplementary material for: Differential and longitudinal immune gene patterns associated with reprogrammed microenvironment and viral mimicry in response to neoadjuvant radiotherapy in rectal cancer
Source: J Immunother Cancer. 2021 Mar 7;9(3):e001717. doi: 10.1136/jitc-2020-001717 (PMC7939016; doi:10.1136/jitc-2020-001717)
Supplement: Supplementary data [file jitc-2020-001717supp001.pdf]

Differential and longitudinal immune gene patterns associated with reprogrammed microenvironment and viral mimicry in response to neoadjuvant radiotherapy in rectal cancer

Authors:
A.C. Wilkins,[1,2$] E Fontana,[3$] G Nyamundanda,[3] C Ragulan,[3] Y Patil,[3] D Mansfield,[1] J Kingston,[4] F Errington-Mais,[4] D.S. Bottomley,[4] K Von Loga,[3,5] H Bye,[3,5] P Carter, [3,5] E Tinkler-Hundal,[4] A Noshirwani,[4] J Downs,[6] M Dillon,[1] S Demaria,[7] D. Sebag-Montefiore,[4] K.J. Harrington,[1,5] N.P. West, [4*] A.A. Melcher,[1,5*] A Sadanandam[3*,#]

Affiliation List

1. Division of Radiotherapy and Imaging, The Institute of Cancer Research, London, United Kingdom.
2. The Francis Crick Institute, London, United Kingdom.
3. Division of Molecular Pathology, The Institute of Cancer Research, London, United Kingdom.
4. Leeds Institute of Medical Research at St. James's, University of Leeds, Leeds, United Kingdom.
5. The Royal Marsden Hospital, London, United Kingdom.
6. Division of Cancer Biology, Institute of Cancer Research, London, United Kingdom.
7. Department of Radiation Oncology and Pathology and Laboratory Medicine, Weill Cornell Medicine, New York, NY, USA

[$]Joint first authors, *Joint senior authors

# Corresponding author: Dr. Anguraj Sadanandam, Head of Systems and Precision Cancer Medicine, Division of Molecular Pathology, The Institute of Cancer Research, 15 Cotswold Road, Sutton, Surrey, SM2 5NG, United Kingdom
Phone: +44 203 437 6440
Email: anguraj.sadanandam@icr.ac.uk
ORCID: 0000-0001-8485-5150

Supplementary Information

Supplementary Methods:

Immunohistochemistry for CD68 and CD163

After heat-mediated antigen retrieval in 10mM citric acid buffer, pH 6.0, for 10 minutes in a 900-watt microwave, slides were washed under running water. To block endogenous peroxidase activity 100μL of dual endogenous enzyme block was pipetted onto each slide for 5 minutes followed by a rinsing in Tris-buffered saline (TBS) for 5 minutes.

100μL of antibody diluent was applied to all specimens to block nonspecific binding. 100μL of CD163 primary antibody (mouse monoclonal clone immunoglobulin (IgG1)/EDHu-1), diluted at 1:200 was added to each slide. All slides were incubated for 60 minutes after which they were washed in TBS with Tween 20 (TBS-T) (2 x 5 minutes) and TBS (5 minutes). 100μL of horseradish peroxidase conjugated polymer was applied as a secondary antibody to the sections and incubated for 10 minutes, and washed as above. 3, 3′

diaminobenzidine tetrahydrochloride (DAB+) chromogen solution (100μL, 10 minutes) was applied to allow visualisation of the first primary antibody. Slides were washed in running water for 5 minutes to remove unbound DAB+ chromogen. 100μL of double stain block was incubated on the slides for 3 minutes after which it was washed off with TBS-T (2 x 5 minutes) and TBS (5 minutes).

100μL of the antibody CD68 (mouse monoclonal clone IgG1/KP1) diluted at 1:3000 was added to each slide and incubated for 60 minutes at room temperature. Washing steps were performed as mentioned above. Following the washing of the slides, 100μL of a secondary antibody rabbit/mouse link solution was pipetted on for 10 minutes, followed by washing as above. A final 100μL of alkaline phosphatase polymer was applied for 10 minutes and washed as above. The second dye-stain to aid in visualisation of the immunohistochemical reaction, permanent red working solution, was applied to all specimens (100μL, 15 minutes). Following this final staining, the slides were washed in running water for 5 minutes, and counterstained with Mayer's Haematoxylin for 1 minute. The slides were dehydrated on a hotplate at 70ºC for 20 minutes, before being immersed in xylene in preparation of being mounted with cover-slips using DePex mountant (VWR BDH Prolabo).

Supplementary Table 1: Genes upregulated in intermediate responding tumours versus tumours showing a good response (false discovery rate <0.04).

Gene Name
ITGAX
ICAM1
CDH5
TNFSF13B
TLR1
CCL28
ENTPD1
PLAU

Supplementary Table 2: The Molecular Signature Databse (MsigDB's) Investigate Gene Sets of hallmark gene sets - Overlap Results - Enrichment analysis of Chauvin's publication (1)**:** See attached excel spreadsheet

Supplementary Table 3: ARCHS4 table of cell populations. This table shows a statistical comparison of the cell populations in the ARCHS4 tissue database that are enriched in the baseline biopsies of poorly responding tumours versus tumours showing a good response to radiotherapy: See attached excel spreadsheet.

Supplementary Table 4: Gene lists from the ARCHS4 database that were used to define the ten cell populations shown in Figure 5A.

Cell Population (ARCHS4)	Genes
MACROPHAGE	CD53;IL15RA;MSR1;CD274;CD40;CD163;ITGAM;IL1R1;IL1R2;ITGB3;IL24;LILRB2;LILRB3;LILRA5;ICAM1;TLR1;CLEC4A;HCK;FCGR3A;PLAU;SPP1;ITGAX;CCL2;CCL18
ALVEOLAR-LIKE MACROPHAGE	CD53;MSR1;CD274;CD163;ITGAM;ITGA4;IL1R2;LILRB2;LILRB3;ICAM1;TLR1;CLEC4A;HCK;FCGR3A;PLAU;ITGAX;CCL2;CCL18
NEUTROPHIL	CD53;CD274;CSF3R;ITGAM;IL1R1;IL1R2;IL24;LILRB2;LILRB3;LILRA5;ICAM1;TLR1;CLEC4A;HCK;FCGR3A;PLAU;ITGAX;IL6R
GRANULOCYTE	CD53;CD274;ENTPD1;CSF3R;ITGAM;IL1R2;LILRB2;LILRB3;LILRA5;TLR1;CLEC4A;HCK;FCGR3A;ITGAX;IL6R
DENDRITIC CELL	CD53;ENTPD1;ITGAM;ITGA4;IL1R2;LILRB2;LILRB3;TNFSF13B;TLR1;CLEC4A;HCK;ITGAX;CCL18;IL6R
VASCULAR SMOOTH MUSCLE	PDGFRB;IL11;CDH5;CD274;C1S;IL1R1;PLAU;ITGB3;VEGFC;CCL2;ICAM1;JAM3
PLASMACYTOID DENDRITIC CELL	CD53;TLR1;IL15RA;HCK;CD274;CD40;LILRB2;LILRB3;LILRA5;TNFSF13B;ICAM1
KUPFFER-LIKE CELL	PDGFRB;IL15RA;C1S;IL1R1;ITGB3;ITGA1;VEGFC;CCL2;ICAM1;JAM3
FIBROBLAST	PDGFRB;IL11;C1S;IL1R1;PLAU;ITGB3;ITGA1;VEGFC;CCL2;JAM3
STROMAL CELL	PDGFRB;IL11;C1S;IL1R1;PLAU;ITGB3;VEGFC;CCL2;JAM3

Supplementary Table 5: Human Gene Atlas table of cell populations. This table shows a statistical comparison of the cell populations in the Human Gene Atlas tissue database that are enriched in the baseline biopsies of poorly responding tumours versus tumours showing a good response to radiotherapy.

See attached excel spreadsheet

Supplementary Table 6: Gene lists from the Human Gene Atlas tissue database that were used to define the three cell populations shown in Figure 5B.

Cell Population (Human Gene Atlas)	Genes
CD14+_Monocytes	CLEC4A;HCK;CD163;ITGAX;LILRB2;LILRB3;LILRA5
CD33+_Myeloid	CLEC4A;HCK;CD163;ITGAM;LILRB2;TYK2;LILRB3;IL6R
SmoothMuscle	IL11;IL24;VEGFC;CCL2;ICAM1

Supplementary Figure 1: A. Boxplot of mean expression of the 40 genes expressed in the poor responding samples in high and low expressors of the third validation cohort samples. **B**. Heatmap to show individual gene expression in the third validation cohort (based on 40 upregulated genes in our study cohort).

See separate pdf.

Supplementary Figure 2: Heatmap to show 198 genes with a significant difference in gene expression between matched pre- and post-radiothearpy tumours showing a good response to radiotherapy.

Supplementary Figure 3: Boxplots to show immune enrichments scores (as per Rooney et al. (2)) in pre-radiotherapy biopsies and post-radiotherapy resection specimens for **A:** Co-inhibition of T-cell scores, **B:** type I IFN scores and **C:** macrophage scores

References

1. Chauvin A, Wang CS, Geha S, Garde-Granger P, Mathieu AA, Lacasse V, et al. The response to neoadjuvant chemoradiotherapy with 5-fluorouracil in locally advanced rectal cancer patients: a predictive proteomic signature. Clin Proteomics. 2018;15:16.
2. Rooney MS, Shukla SA, Wu CJ, Getz G, Hacohen N. Molecular and genetic properties of tumors associated with local immune cytolytic activity. Cell. 2015;160(1-2):48-61.
