# Supplementary figures and images for: Differential and longitudinal immune gene patterns associated with reprogrammed microenvironment and viral mimicry in response to neoadjuvant radiotherapy in rectal cancer
Source: J Immunother Cancer. 2021 Mar 7;9(3):e001717. doi: 10.1136/jitc-2020-001717 (PMC7939016; doi:10.1136/jitc-2020-001717)

### Supplementary Figure 1

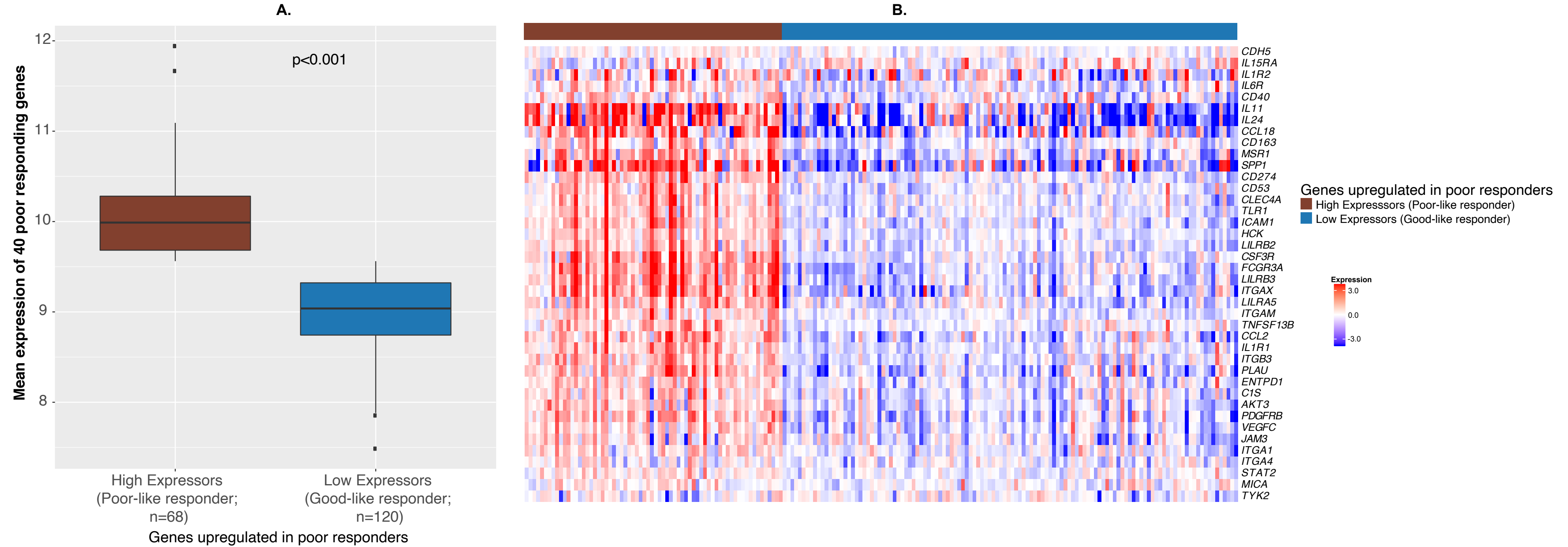

Supplement: Supplementary data [file jitc-2020-001717supp003.pdf]

## Supplementary Figure 2

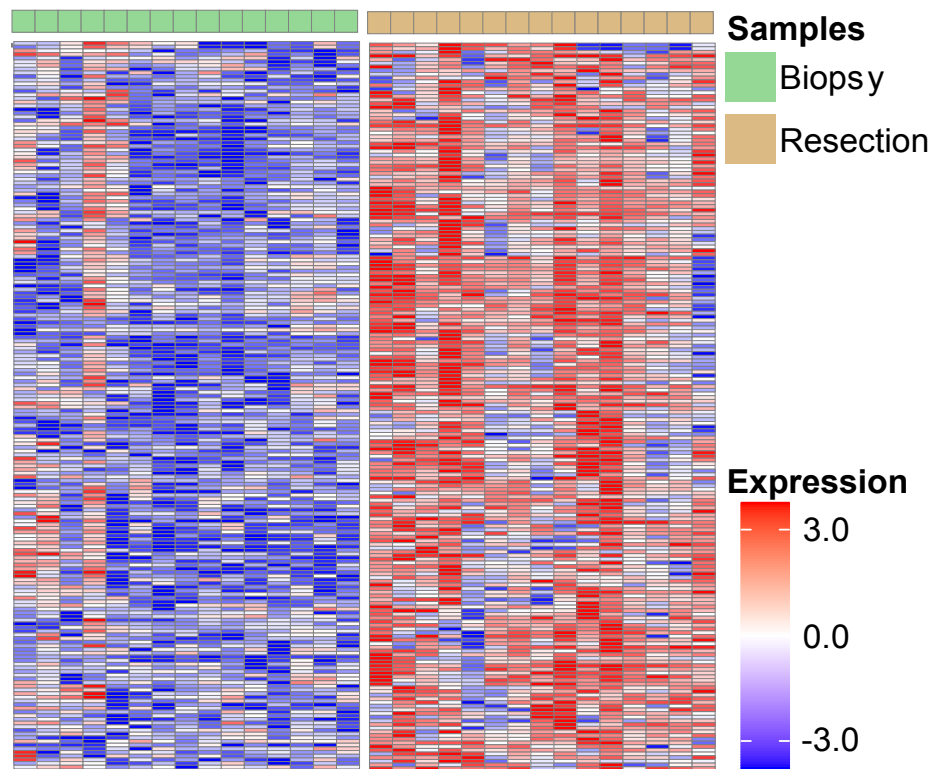

Supplement: Supplementary data [file jitc-2020-001717supp006.pdf]

Supplementary Figure 3

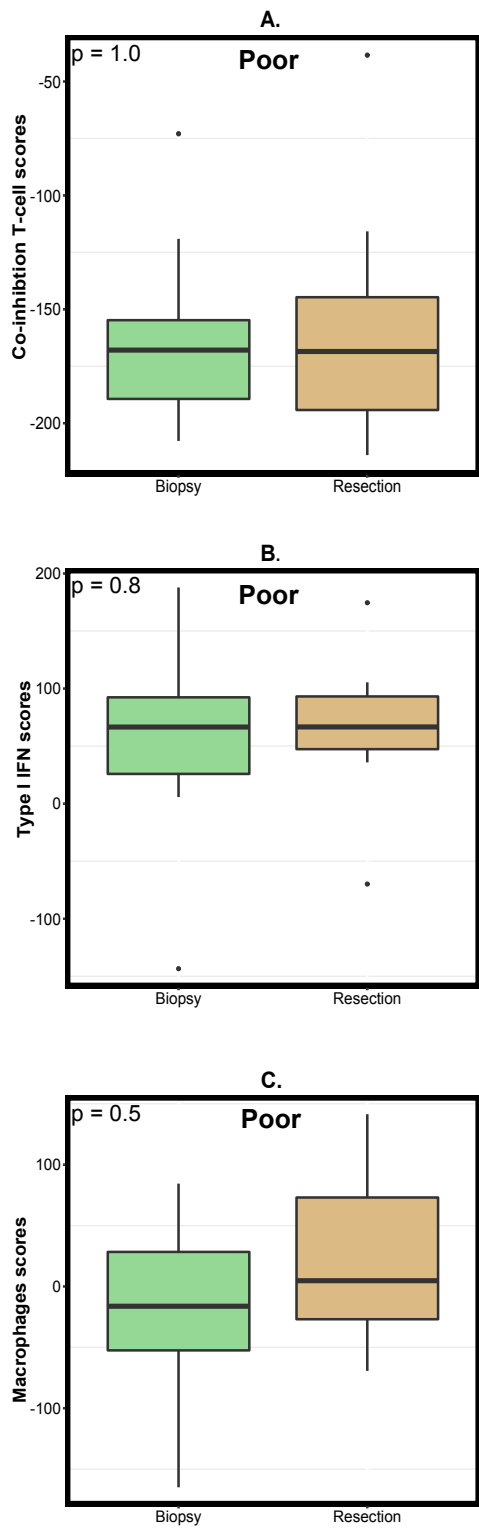

Supplement: Supplementary data [file jitc-2020-001717supp007.pdf]
